# How Can Overlooking Social Interactions, Space Familiarity or Other “Invisible Landscapes” Shaping Animal Movement Bias Habitat Selection Estimations and Species Distribution Predictions?

**DOI:** 10.1002/ece3.70782

**Published:** 2025-01-08

**Authors:** Romain Dejeante, Rémi Lemaire‐Patin, Simon Chamaillé‐Jammes

**Affiliations:** ^1^ CEFE Univ Montpellier, CNRS, EPHE, IRD Montpellier France; ^2^ ISPA Bordeaux Sciences Agro, INRAE Villenave d'Ornon France; ^3^ Department of Zoology and Entomology Mammal Research Institute, University of Pretoria Pretoria South Africa

**Keywords:** habitat selection, RSF, SDM, social environment, spatial memory, species distribution

## Abstract

Species' future distributions are commonly predicted using models that link the likelihood of occurrence of individuals to the environment. Although animals' movements are influenced by physical and non‐physical landscapes, for example related to individual experiences such as space familiarity or previous encounters with conspecifics, species distribution models developed from observations of unknown individuals cannot integrate these latter variables, turning them into ‘invisible landscapes’. In this theoretical study, we address how overlooking ‘invisible landscapes’ impacts the estimation of habitat selection and thereby the projection of future distributions. Overlooking the attraction towards some ‘invisible’ variable consistently led to overestimating the strength of habitat selection. Consequently, projections of future population distributions were also biased, with animals following changes in preferred habitat less than predicted. Our results reveal an overlooked challenge faced by correlative species distribution models based on the observation of unknown individuals, whose past experience of the environment is by definition not known. Mechanistic distribution modeling integrating cognitive processes underlying movement should be developed.

## Graphical abstract

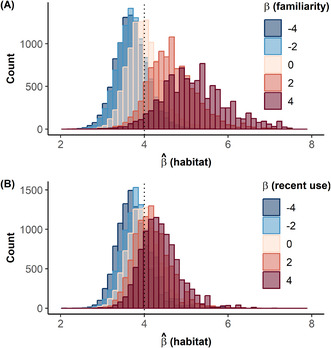

## 1 Introduction

Moving from descriptive to predictive ecology has been a key endeavor for ecologists for decades, especially as the demand for applied science increased. For instance, understanding and predicting the distribution of animals, especially in changing environments, is considered critical to developing appropriate conservation and management practices (Sofaer et al. 2019). However, as highlighted by Mouquet et al. (2015), predictions can “grow faster than our understanding of ecological systems”, and there is always a great need to try to reduce structural uncertainty of predictive models, among the other sources of uncertainty, as much as possible (Elith, Burgman, and Regan 2002).

Species distribution models (SDMs) are now commonly used to predict species' habitat suitability by comparing the environmental characteristics of locations where the individuals have been observed to a set of available locations. Such models can be developed from snapshot observation data of unknown individuals, in which case the models are generally termed SDMs based on presence/pseudo‐absence data (Elith and Leathwick 2009) and estimate “habitat suitability”, or from animal tracking data, in which case the models are generally termed resource selection functions (RSF) on used/available data (Boyce et al. 1999) and estimate “habitat selection”. The difference in terminology hides that the class of models used on the two different types of data are generally similar (Matthiopoulos et al. 2015). Although fitted at different spatial scales—RSFs usually modeling within‐home‐range habitat selection resulting from the behavioral decisions of an animal and SDMs usually predicting species distribution at much larger, sometimes up to worldwide, scales—these models fundamentally estimate the disproportionate presence of the species in some habitats.

Habitats are generally defined as “a point in environmental space, a space whose dimensions are environmental variables (conditions, resources, and risks)” (Matthiopoulos, Fieberg, and Aarts 2023). For instance, a habitat will be characterized by having a specific vegetation type, specific slope, and being at a specific distance to some distinct features of the landscape (e.g., roads or water sources). However, an animal's habitat selection is not solely influenced by characteristics of locations across the set of environmental layers that are easily observable or measurable by a human analyst but also by variables related to current or past predators' presence, a key component of the landscape of fear (Laundre, Hernandez, and Ripple 2010), to the social landscape (Webber and Vander Wal 2018; Armansin et al. 2020), or to the memory landscape (Smouse et al. 2010). Although the importance of these landscapes on animal movement is increasingly recognized by ecologists, especially regarding the influence of predator–prey interactions and spatial memory, their estimation requires long‐term tracking of animals (e.g., to detect which locations are familiar for each individual) and intensive tracking of many individuals (e.g., to detect the encounters between preys and predators). Therefore, species distribution predicted from RSFs based on tracking data rarely accounts for these processes, commonly pooling tracked animals together and ignoring individual‐level information (Chambault et al. 2021; McCabe et al. 2021). Similarly, SDMs developed from snapshot observations and species‐level occurrence records will necessarily fail to integrate the influence of these processes that need individual‐level information to be estimated. Because of the difficulty for researchers to measure and account for these landscapes, which are relevant for animals but do not map directly with physical features of the environment, we termed them “invisible landscapes”.

One might, however, expect that overlooking the influence of these “invisible landscapes” on animals' movement decisions may severely impair the accuracy of predictive distribution models. Indeed, exhaustively describing the variables that influence animals' movement decisions is critical to increasing the performance of species distribution models (Zeller et al. 2017). Also, the lack of one variable in the species distribution model may bias the estimation of the effect of others (Van Moorter et al. 2013). Although many ecologists are aware of the importance of non‐physical landscapes (e.g., landscape of fear, social landscape, memory landscape) (Fagan et al. 2013; Laundre, Hernandez, and Ripple 2010; Webber et al. 2022), most predictions of species distributions fail to integrate their influence, and there is a great need to understand whether systematic biases in predictions are to be expected.

Here we contribute a theoretical study to highlight how accounting, or not, for “invisible landscapes” affects species distribution models. We used an individual‐based model to simulate the movement and distribution of animals whose habitat selection was known and influenced by space familiarity (i.e., whether a location had been used before by the individual) and recent use by conspecifics. We then investigated to what extent (1) the estimation of the strength of selection for some habitat variables and (2) the prediction of population distribution could be biased when the influence of familiarity and recent use were not accounted for in the analysis. Because SDMs are often used to predict species distribution in changing environments or in future conditions, we used our theoretical approach to test how “invisible landscapes” could bias the prediction of population distribution in such situations.

## 2 Material & Methods

### 2.1 Testing the Effect of Familiarity and Recent Use by Conspecifics on Habitat Selection Estimation

To test the effect of not accounting for familiarity and recent use of the landscape by conspecifics when estimating habitat selection, we (1) simulated the movements of animals with known selection coefficients for habitat type, familiarity, and level of recent use, and (2) compared the “true” coefficient of habitat selection with the one estimated from models that did not contain familiarity and recent use levels as predictors.

#### 2.1.1 Description of Landscape, Movement Model, and Familiarity and Recent Use Metrics

We developed a model that, for each run, simulates the movements of 500 individuals over a 100 × 100 habitat raster map during 1000 time steps. The movement of each animal was itself generated using the local Gibbs (LG) movement model proposed by Michelot, Blackwell, and Matthiopoulos (2019). The LG model is a step selection model that ensures that the utilization distribution of the animal is consistent with a predefined RSF whose coefficients are used to parametrized the LG model. For each time *t*, a random location was sampled within a 5‐cell radius around the current location, 100 potential locations were then uniformly generated within the 5‐cell radius around this random location, and the location at time *t + 1* was sampled among them with probabilities proportional to the RSF score. In this work, the RSF used to simulate the movement of individuals was as follows:

(1) $$w\left(c\right)=\exp\left(\beta_{\text{habitat}}h\left(c\right)+\beta_{\text{familiarity}}f\left(c\right)+\beta_{\text{recent}\;\mathrm{use}}r\left(c\right)\right)$$

with *w*(*c*) the RSF score for the cell *c* and where *h*(*c*), *f*(*c*), and *r*(*c*) describe the values of habitat, familiarity, and recent use variables—described below—for the cell *c*. Habitat, familiarity, and recent use are variables affecting the movement of the animals, with predetermined coefficients of selection—here referred to with *β*—that were varied, between simulations, from negative (i.e., simulating avoidance/repulsion) to positive (i.e., simulating selection/attraction) values. For all variables, we used a gradient of values ranging from −4 to 4 by 0.5 increments.

##### 2.1.1.1 “Habitat” Map

We assumed that two habitat types existed in the landscape. To create the habitat raster map, and for each replication of the landscape, we did as follows: Using the localGibbs package (Michelot, Blackwell, and Matthiopoulos 2019), we first generated over the extent of the map a spatially correlated Gaussian random field (with a defined spatial autocorrelation *ρ* = 10) whose values, ranging between 0 and 1, were then attributed to each cell. We then used a threshold value *h* to discretize these continuous values into binary ones, so that 1 (respectively 0) represented the presence of habitat A (respectively B) in the cell. Thus, *h* also defined the proportion of habitat A in the landscape. By default, we used *h* = 0.5. We initially tested the influence of the patchiness of the landscape by using two different values of spatial correlation when generating the Gaussian random field (see Figure S1 for illustration). Results were similar, and we therefore present here results obtained for the patchier landscapes.

##### 2.1.1.2 “Familiarity” Map

Following previous works (Van Moorter et al. 2013; Wolf et al. 2009), we modeled familiarity as a binary raster map, with 0 (respectively 1) indicating an unfamiliar (respectively familiar) cell. During simulations, each individual had its own familiarity map, which was initialized at 0 for all cells. Thus, at the beginning of each simulation, individuals were assumed to be unfamiliar with the landscape. Then, at each time step, the familiarity maps of all individuals were updated, with visited cells shifting to a value of 1. For simplicity, we assumed that individuals did not forget whether a previous location had previously been visited or not.

##### 2.1.1.3 “Recent Use” Map

During each simulation, the level of recent use of cells was tracked through one dynamic raster map, which was shared by all individuals of a simulation. At the beginning of the simulation, the map of recently used areas had cell values that were all 0. Then, at each time step, once all individuals had relocated, we updated the map of recently used areas by calculating, for each cell, the number of times the cell had been visited by at least one individual over the last 20 time steps. This map can therefore be seen as storing an index of the recent presence of conspecifics. We used a temporal window since (1) animals may be able to detect only the recent, not the older, use of a location by other animals, such as from mark scents, and (2) since resources may regenerate after a long enough time period.

#### 2.1.2 Estimation of Habitat Selection Coefficients From Simulated Movements

We used the model presented above to investigate to what extent selection for familiar or recently used locations could influence, and in particular possibly bias, the estimation of the strength of habitat selection when they are not taken into account.

We did so by, firstly, running 20 replications of the model for each combination of values of the selection coefficient for habitat, familiarity, and recent use predictors. Secondly, we fitted, to each movement trajectory (i.e., 20 replications × 500 individuals), an RSF with habitat as the sole predictor to obtain the estimate coefficient of selection for habitat ${\hat{\beta }}_{\text{habitat}}$ when familiarity and recently used areas were not known or taken into account. To do so, we calculated the selection ratio of each habitat ${\mathrm{SR}}_{A}$ (respectively ${\mathrm{SR}}_{B}$) as the proportion of cells of habitat A (respectively habitat B) among the used locations, divided by the proportion of cells of habitat A (respectively habitat B) among all cells of the landscape, that is, the available locations. The habitat selection coefficient that would be estimated from an RSF with habitat as the sole predictor is given by (Equation 2).

(2) $${\hat{\beta }}_{\text{habitat}}=\log\left(\frac{{\mathrm{SR}}_{A}}{{\mathrm{SR}}_{B}}\right)$$

Lastly, we compared the estimated selection coefficient from the RSF (${\hat{\beta }}_{\text{habitat}}$) with the theoretical values in the simulation ($\beta_{\text{habitat}}$), and investigated whether the difference between the two was important and varied consistently with the values of $\beta_{\text{familiarity}}$ and $\beta_{\text{recent}\;\mathrm{use}}$.

As we expected consequences on predictions of animal distribution (see Section 2.2), we wanted to explore through which mechanism space familiarity and recent use of areas could impact these distributions. We focused on the movement rate of individuals, calculated here as the net displacement over 10 time steps, from simulations conducted in Section 2.1.1. We used the average movement rates of individuals moving independently of familiarity and recent use (but with the same strength of habitat selection) as a reference value. We then compared, for all combinations of values for the selection coefficients for familiarity and recent use predictors, the ratio between the average movement rates observed and the reference value.

### 2.2 Testing the Effect of Familiarity and Recent Use on Population Distribution Predictions

As we found that not accounting for familiarity and recent use bias the estimation of habitat selection (see Section 3.1), we studied to what extent this bias could lead to errors in the estimation of the distribution of the individuals in a changing environment.

First, to simulate a changing environment, we combined spatially correlated Gaussian random fields with planar gradient neutral landscapes to generate replicated series of four habitat maps that had an increasing proportion of the selected habitat along the West–East axis, which could therefore be seen as an axis of “good” habitat expansion. Each series was assumed to represent one changing landscape at four arbitrary times referred to as T0, T1, T2, and T3 (see Figure S1 for an illustration of one series).

Second, for each combination of coefficients for habitat type ($\beta_{\text{habitat}}$), familiarity ($\beta_{\text{familiarity}}$) and recent use ($\beta_{\text{recent}\;\mathrm{use}}$), we ran simulations on landscape series, simulating the movement of 500 individuals over 500 time‐steps in each landscape (T0–T3). At the start of the simulations (at T0), individuals were randomly located within the western area (position on the *x*‐axis ≤ 10), where habitat A was more common. Then, their locations at the end of T0, as well as space use familiarity and recent use maps, were used at the beginning of T1 simulations, and so on. Simulations followed four scenarios differing by how animal movement was simulated:

- *Reference Scenario* The movement of animals was based on a fully parametrized LG movement model whose coefficients were those used in Section 2.1 $\left(\beta_{\text{habitat}},\beta_{\text{familiarity}},\beta_{\text{recent}\;\mathrm{use}}\right)$. This scenario was assumed to represent “the truth”.
- *Scenario 1* The movement of animals was influenced only by habitat type, based on an LG movement model whose coefficient for habitat type (${\hat{\beta }}_{\text{habitat}}$) had been estimated in Section 2.1 by fitting a habitat‐only RSF model to movement data obtained by simulations with a fully parametrized LG model. Distributions emerging from this scenario are those that ecologists would often generate as they emerge from the standard approach of making predictions from estimations of habitat selection coefficients without accounting for “invisible landscapes”.
- *Scenario 2* The movement of animals was based on a fully parametrized LG model, as in the reference scenario, but with the coefficient for habitat type being the one estimated from a habitat‐only RSF model, as in Scenario 1 $\left({\hat{\beta }}_{\text{habitat}}\beta_{\text{familiarity}}\beta_{\text{recentuse}}\;\right)$. This scenario allowed us to test to what extent including correct information on the effect of “invisible landscapes” allowed us to correct prediction errors induced by a biased estimation of selection for habitat type.
- *Scenario 3* The movement of animals was based on a habitat‐only LG model, with the coefficient for habitat type being the correct one $\left(\beta_{\text{habitat}}\right)$. This scenario allowed us to test to what extent overlooking “invisible landscapes” can induce errors in predictions if selection for habitat type has been correctly estimated.

For all scenarios, simulations were replicated 20 times, and for all simulations, we estimated at time T0, T1, T2, and T3 the density of animal locations across the West–East axis of “good” habitat expansion. This allowed us to describe the changes in the distribution of the population in the landscapes. To compare the distributions emerging from Scenarios 1 to 3 with the ones from the reference scenario, we used the index proposed by Pastore and Calcagnì (2019) to measure the overlap between the distributions (see Figure S2 for an illustration of such overlapping distributions). The index takes greater values as overlap increases, up to a maximum value of 1 when the compared distributions are exactly the same.

## 3 Results

### 3.1 Influence of “Invisible Landscapes” on Habitat Selection Analysis

**FIGURE 1:**
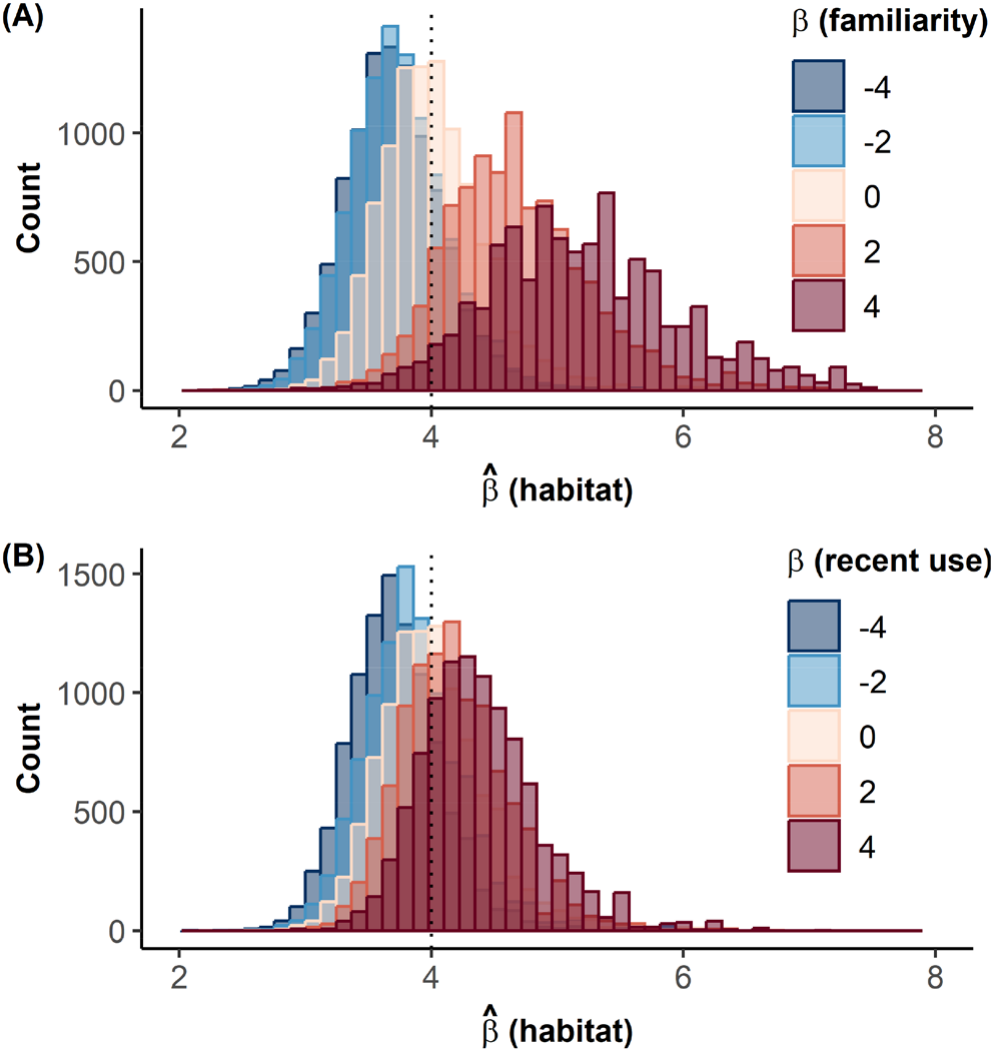
Influence of the strength of selection for familiar space ($\beta_{\text{familiarity}}$) (A) and for recently used areas ($\beta_{\text{recentuse}}$) (B) on the estimation of the strength of selection for habitat type (${\hat{\beta }}_{\text{habitat}}$). The dotted vertical lines indicate the value of $\beta_{\text{habitat}}$ used in the simulations. The distribution of the estimates ${\hat{\beta }}_{\text{habitat}}$ across varying values of $\beta_{\text{familiarity}}$ and $\beta_{\text{recentuse}}$ are shown along a blue (i.e., negative strength) to red (i.e., positive strength) gradient. The influence of space familiarity on habitat selection estimations is shown for null effect of recent use variables ($\beta_{\text{recentuse}}=0$) (A) and inversely ($\beta_{\text{familiarity}}=0$) (B). Simultaneous influences of familiarity and recent use variables are provided in Figure S2.

**FIGURE 2:**
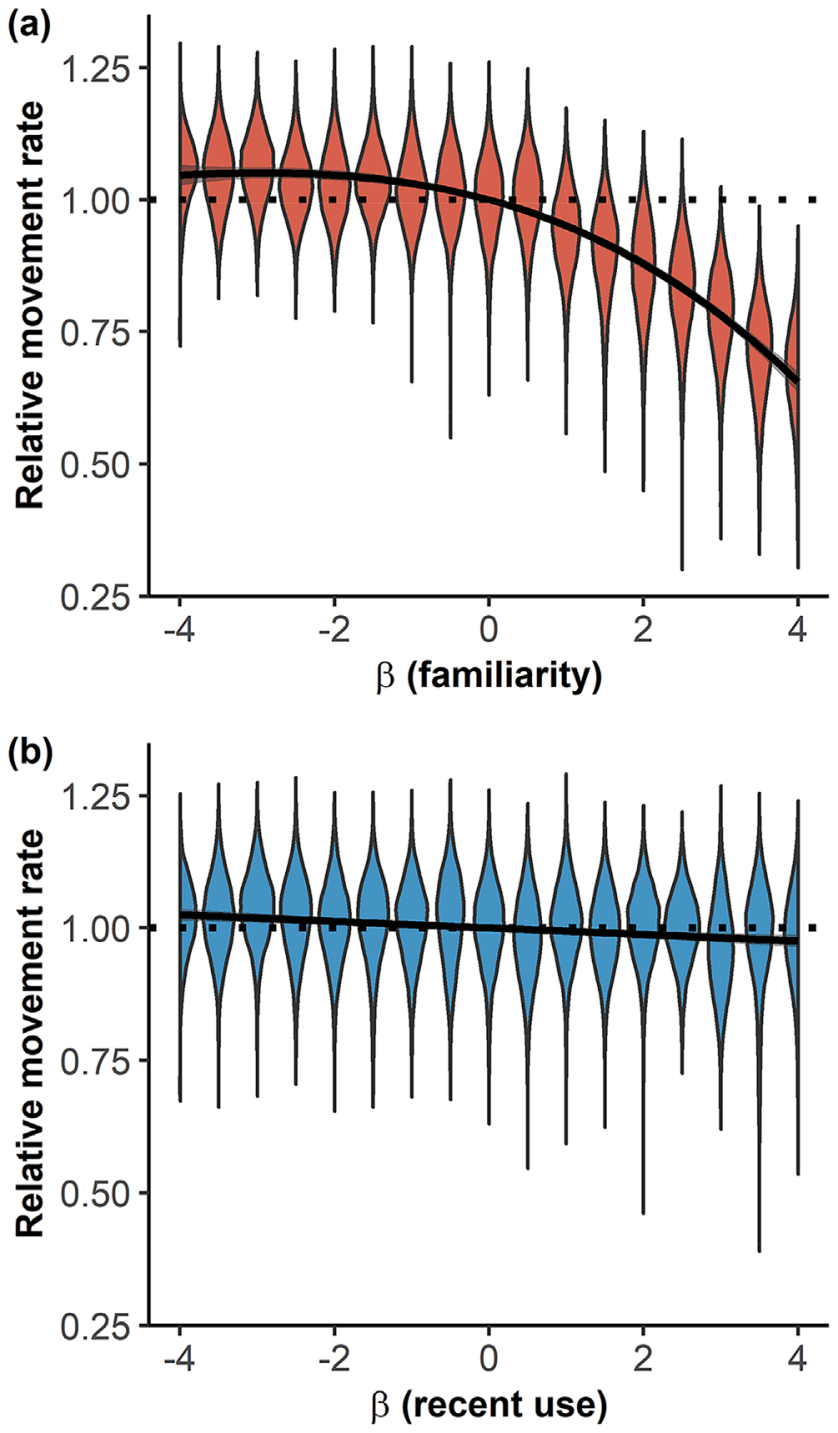
Influence of the strength of selection for familiar space ($\beta_{\text{familiarity}}$) (A) and for recently‐used areas ($\beta_{\text{recentuse}}$) (B) on the movement rate of simulated individuals, relative to the average movement rate of individuals with the same selection strength for habitat type but with null selection for familiar space and recently used areas. Violin plots show the distribution of movement rates obtained over 20 habitats × 500 replications for each set of habitat selection coefficients for familiar and recent use habitats.

Estimating the strength of habitat selection (${\hat{\beta }}_{\text{habitat}}$) from a habitat‐only model clearly provided biased results when the animal movement was driven by habitat selection but also other variables such as familiarity (Figure 1A) or recent use (Figure 1B). Importantly, habitat selection was estimated to be stronger than it actually was if the animal tended to select for greater value of the overlooked variables, that is, if the selection coefficients for these variables were positive. The opposite held true for negative coefficients. The bias increased when the influence of these overlooked variables on movement increased (i.e., when coefficients were greater). The biases associated with each overlooked variable could sometimes compensate each other, as habitat selection strength was correctly estimated for specific combinations of non‐null $\beta_{\text{familiarity}}$ and $\beta_{\text{recent}\;\mathrm{use}}$ (see Figure S2). Note that, when the animal's movement was not influenced by familiarity or recent use, we could correctly recover, with some noise due to the stochastic nature of simulations, the theoretical coefficient of habitat selection used in the model. This was expected but demonstrated the validity of the approach. In addition to the bias observed in the estimation of the strength of selection for habitat variables, individuals selecting familiar habitats exhibited smaller movement rates than individuals moving independently of familiarity (Figure 2A). In contrast, we did not find any influence of the selection, nor the avoidance, of recently used habitats on the movement rate of the simulated individuals (Figure 2B).

### 3.2 Influence of “Invisible Landscapes” on Population‐Level Inference

**FIGURE 3:**
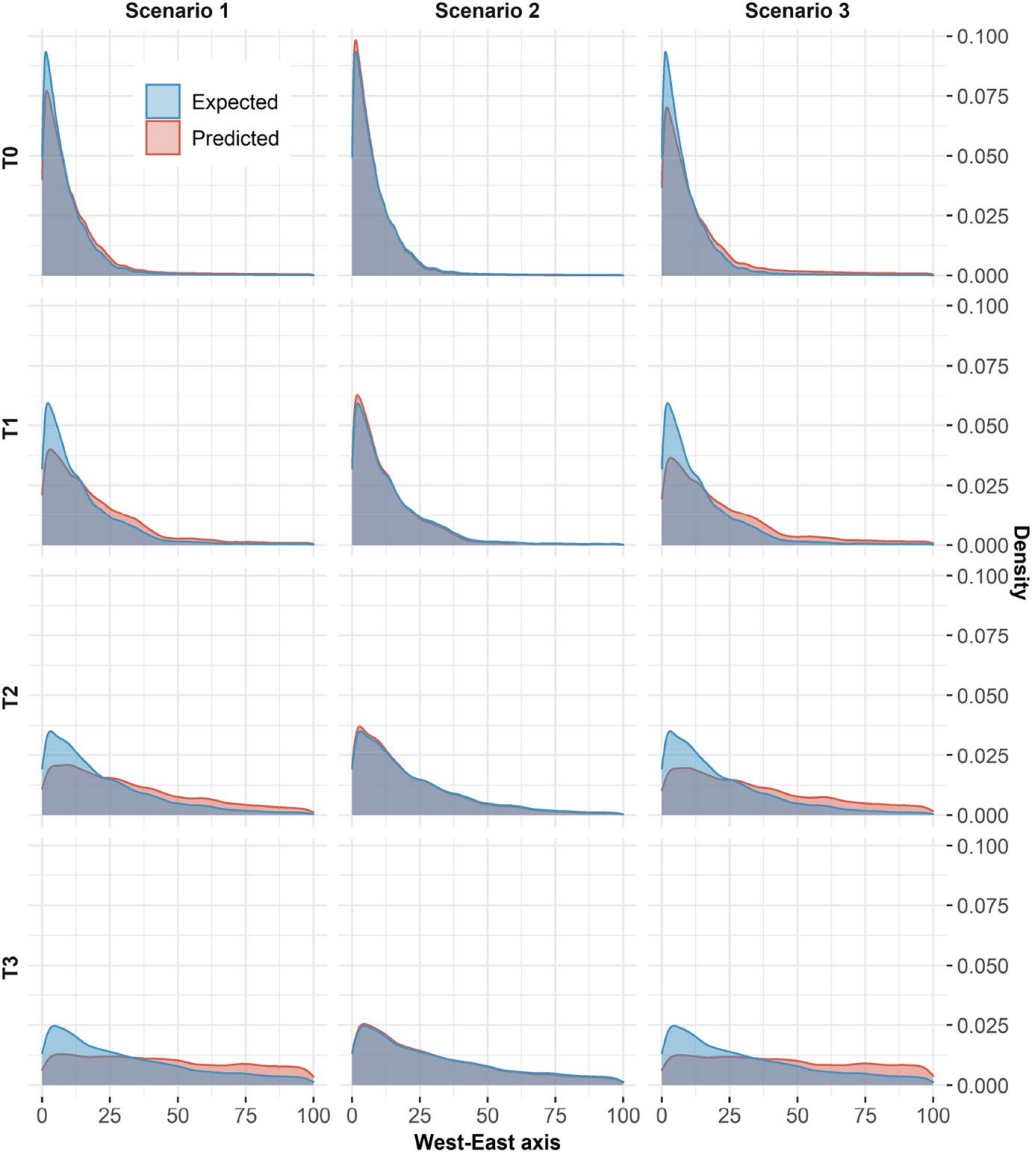
Comparison between the expected (reference Scenario; blue) and the predicted (Scenarios 1; 2; 3; red) distributions of individuals along a habitat‐expanding gradient (West–East axis) at time T0, T1, T2, T3. The expected distribution was obtained by simulating individuals with a fully parametrized LG movement model ($\beta_{\text{habitat}}=4;\beta_{\text{familiarity}}=2;\beta_{\text{recentuse}}=0$). Scenario 1: Distribution predicted from the biased estimate of habitat selection (${\hat{\beta }}_{\text{habitat}}=4.7;\beta_{\text{familiarity}}=0;\beta_{\text{recentuse}}=0$). Scenario 2: Distribution predicted from biased estimate of habitat selection and the selection coefficients for familiar and recently used areas (${\hat{\beta }}_{\text{habitat}}=4.7;\beta_{\text{familiarity}}=2;\beta_{\text{recentuse}}=0$). Scenario 3: Distribution predicted from the coefficient of habitat selection only ($\beta_{\text{habitat}}=4;\beta_{\text{familiarity}}=0;\beta_{\text{recentuse}}=0$).

**FIGURE 4:**
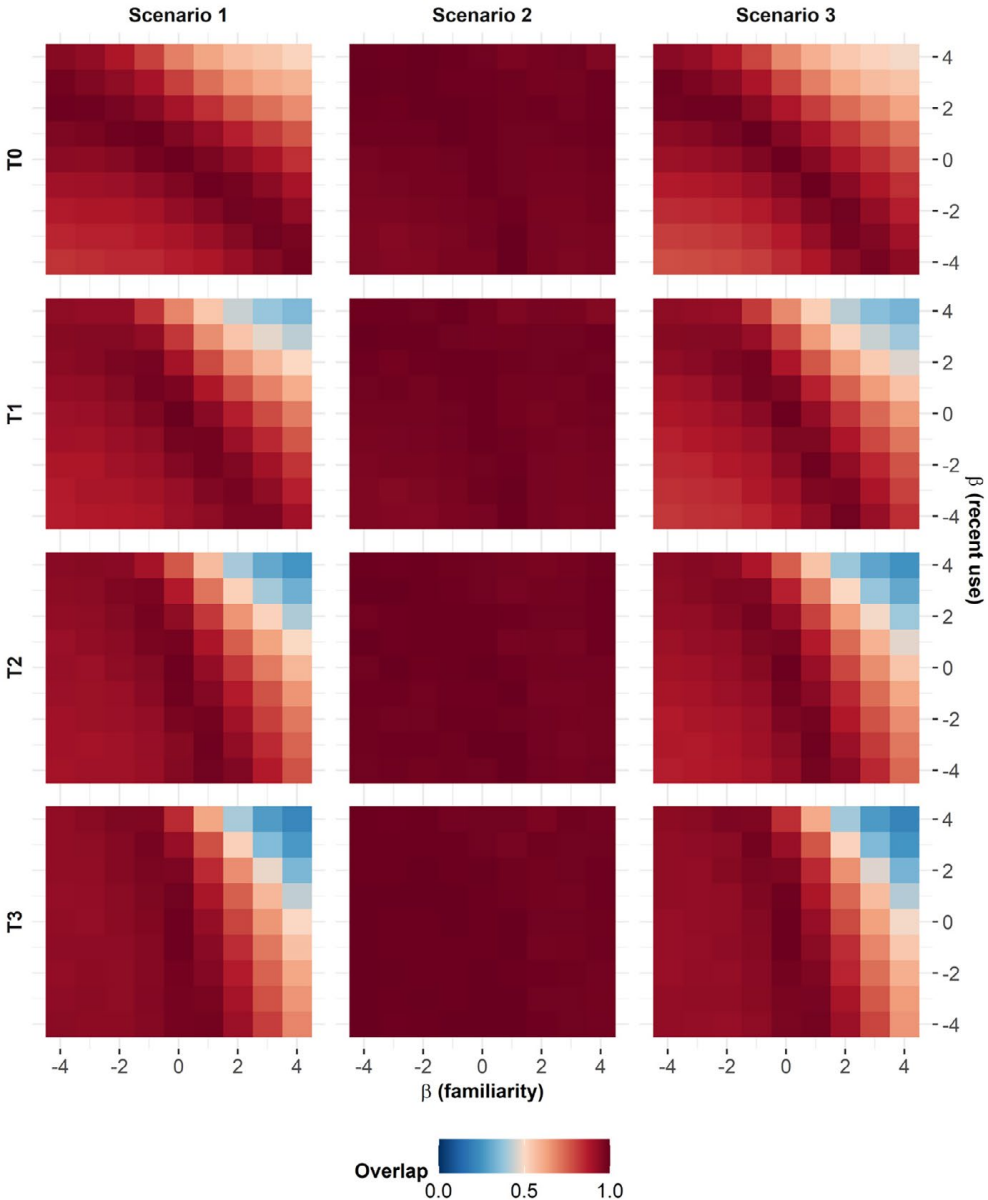
Overlap between the predicted and expected population distributions along a habitat‐expanding gradient (T0–T4) according to the individual selection coefficient for familiar space $\left(\beta_{\text{familiarity}}\right)$ and recently used areas ($\beta_{\text{recentuse}}$) used in the expected distribution case. Scenario 1: Population distributions predicted from the estimated coefficient of habitat selection $\left({\hat{\beta }}_{\text{habitat}}\right)$. Scenario 2: Population distributions predicted from the estimated coefficient of habitat selection and the selection coefficients for familiar and recently used habitats $\left({\hat{\beta }}_{\text{habitat}},\beta_{\text{familiarity}},\beta_{\text{recentuse}}\right)$. Scenario 3: Population distributions predicted from the actual coefficient of habitat selection $\left(\beta_{\text{habitat}}\right)$.

Predictions of the distribution of individuals in a changing landscape, when made using models previously fitted with only habitat as a predictor, were increasingly wrong over time (Figures 3 and 4; Scenario 1). This was particularly true when the selection of animals for familiar areas was important and, to a lesser extent, when the selection for areas recently used by conspecifics was also important (Figure 4). In any case, the error made was a consistent bias towards an overestimation of the tracking of the change in the distribution of the “good” habitat by the population when individuals selected for familiar locations (Figure 3). Conversely, we would underestimate the tracking of the change of the distribution of the “good” habitat by the population when individuals avoid familiar or recently used locations (Figure S2).

Somewhat unexpectedly, predictions made with models that had the correct selection coefficients for familiarity and recent use variables, but for which the coefficient for the habitat type variable had been estimated from habitat‐only models as in Scenario 1, were most often “good” (Figure 4; Scenario 2). Predictions made with models that had the correct selection coefficient for habitat type but did not include familiarity or recent use variables were often wrong (Figure 4; Scenario 3), in a way similar to what was observed in Scenario 1.

## 4 Discussion

In this study, we used computer simulations to show that overlooking “invisible landscapes”, that is, non‐physical variables that are relevant for animals (here social and memory landscapes) but difficult to grasp and measure for the human analyst, induces bias in habitat selection analyses and population‐level projections. These results are important because an increasing number of empirical studies are confirming what had long been “known” by field ecologists, that the attractions to familiar sites (Merkle, Fortin, and Morales 2014; Merkle et al. 2019; Ranc et al. 2020, 2021; Wolf et al. 2009) or to social mates (Buxton et al. 2020; Peignier et al. 2019; Webber et al. 2024) are indeed important, if not critical, in driving animals' movement decisions.

### 4.1 How to Integrate “Invisible Landscapes” in Habitat Selection Analyses?

Of the “invisible landscapes” that affect the habitat choices of animals, not all present the same challenges to the human analyst. In some rare instances, enough social partners, competitors, and predators can be tracked by GPS to evaluate the social landscape or the landscape of fear in which animals move (Wilmers et al. 2015). However, even when a whole group is tracked—which is rarely possible, but see Papageorgiou, Nyaguthii, and Farine (2024) or Strandburg‐Peshkin et al. (2015)—researchers “are blind to the absence/presence of untracked individuals in close spatial proximity” (He et al. 2023). Supplementing GPS tracking with observational data—for example, using animal‐borne video collars to evaluate encounters with untracked individuals (Andersen et al. 2020; Dejeante, Valeix, and Chamaillé‐Jammes 2024b; Tremblay et al. 2014)–could help, but it remains an uncommon approach to make visible the presence of untracked individuals. In addition to gaining information about the current presence of social mates, competitors, or predators, gaining information about the past presence of some individuals at a particular site remains another challenge for ecologists. Even the spatial familiarity of tracked individuals is generally unknown before individuals are equipped with a GPS tracking device, except in the special case of reintroductions (Berger‐Tal and Saltz 2014; Ranc, Cagnacci, and Moorcroft 2022). As tracking duration increases, one can, however, start considering the familiarity of individuals with the visited locations, and this factor has been increasingly integrated into habitat selection models recently (Kim et al. 2023; Merkle et al. 2019; Oliveira‐Santos et al. 2016; Ranc et al. 2020). Unfortunately, animals' decisions *in nature* are likely driven by a number of these non‐physical variables, often turning them into “invisible landscapes” because of their difficulty to grasp and measure.

Here, our results reveal strong, consistent biases produced by failing to account for “invisible landscapes” in RSFs, and it therefore calls for integrating as much as possible of the factors that drive animals' movement decisions. Overlooking the attraction of animals towards “invisible landscapes”, for instance familiar space, consistently led to an overestimation of the strength of habitat selection. This result arose from a positive feedback loop that makes animals overuse “good” habitats: by being more familiar with “good” habitats (because of a preliminary habitat selection), animals that randomly select a location among the set of familiar locations are more likely to draw a location from “good” habitats. Here, however, because we simulate neither the fact that animals could preferentially remember “good” rather than “bad” quality habitats nor the fact that they can forget previously visited sites, our results do not inform on the strength of the bias induced by overlooking the attraction towards familiar space during analysis but clearly expose the direction of the bias (i.e., it inflates positive selection coefficients even higher). Similar results—and feedback loops—were obtained regarding the “recent use” variable: overlooking the avoidance of recently used areas led to underestimations of an animal's habitat selection. Conversely, overlooking the attraction towards locations recently used by conspecifics led to overestimations of the strength of habitat selection. Interestingly, the reverse mechanism had been described by Van Moorter et al. (2013), showing how one can estimate a spurious selection for familiar places that is the selection for a physical variable lacking from the habitat selection analysis. Together, these results show the importance of exhaustively describing the various “landscapes” on which an animal bases its decision to move when estimating habitat selection. This is, however, likely to be often difficult, if not impossible. In practice, ecologists will never have access to the “true value” of coefficients, and estimations based on actual data will likely always remain biased. We therefore suggest that, if habitat selection coefficients are used in downstream tasks, sensitivity analyses of key outputs to changes in coefficient values, possibly directionally when the direction of the bias is known, should be conducted more routinely. For instance, if one is assessing connectivity from a habitat selection analysis that would not have taken space familiarity into account, we would recommend analyzing the sensitivity of connectivity results to decreases in habitat selection coefficients.

### 4.2 How to Integrate “Invisible Landscapes” in Species Distribution Models?

One key remark we wish to make here is that information about a number of important factors driving animals' decisions about where to move, such as space familiarity, past experience with predators, or more generally any variable related to the past experience of a specific individual, will never be available in studies only based on snapshot (one‐point‐in‐time) observations of species' presence that ignore the identity of the observed individuals. In this case, researchers seeking to model the disproportionate presence of the species in some habitats have no other choice than to overlook the effect of attraction for familiar places—a likely very common process in space use decisions (Merkle, Fortin, and Morales 2014; Merkle et al. 2019; Ranc et al. 2020, 2021; Wolf et al. 2009). SDM studies are sometimes conducted at a very large scale, which may come at the expense of being less able to accurately account for some of the “invisible landscapes” that may be experienced by an animal at finer spatial scales. To what extent that matters, however, remains to be ascertained. In particular, analyses conducted over areas in which accessibility of the various habitats to the animals are not guaranteed may suffer from other, possibly more important biases (Matthiopoulos et al. 2020). Our results may in such situations be less relevant. However, we cannot think of any good reasons why SDM based on species occurrences collected over areas in which all habitats are available to the animals (e.g., when using census data from a national park) would not suffer from the bias we describe here.

Accurately estimating habitat selection is obviously an important step towards unbiased projections of population distribution. However, here, we found that mechanistic distribution modeling integrating the cognitive processes underlying animal movement is, at least, as important as accurately estimating habitat selection. Indeed, our results show that integrating “invisible landscapes” not only when building SDM/RSF explanatory models to obtain coefficients but also when using these models for predictions is crucial to accurately predict population distribution and can even mitigate the consequence of estimating biased coefficients of habitat selection (see Section 3.2). As a result, if SDMs based on snapshot observation of unknown individuals fail to integrate “invisible landscapes” when linking species occurrence to landscape composition, efforts should be made to integrate “invisible landscapes” in the projections made from these models. In particular, our results suggest that the reduction of the movement rate of animals using spatial memory to navigate is an important factor to consider when predicting the distribution of animals over changing conditions. Unfortunately, there does not seem to be any obvious solution to this important issue. The SDM literature has recently seen a surge of methods to account for dispersal and connectivity constraints (Barbet‐Massin, Thuiller, and Jiguet 2012; Boulangeat, Gravel, and Thuiller 2012; Monsimet et al. 2020), which could provide a partial solution to this problem, although ultimately those do not represent directly the process presented here. Integrating past experience will likely require using mechanistic SDM that directly simulates biological processes (as opposed to correlative SDM; sensu Dormann et al. 2018). Thus, we add to the list of reasons (e.g., integration of physiological processes or biotic interaction) that have been called for to move away from correlative SDM. While doing so, we recognize the complexity and challenges of parametrizing mechanistic models but highlight that habitat selection models such as RSF or movement models such as integrated step‐selection functions or LG models are appropriate tools to gain insights about parameters. Ultimately, predictive distribution models should also include the demographic fate of individuals, and the recently developed spatial absorbing Markov chain framework (Fletcher et al. 2019) is an appealing first step towards this goal, of intermediate complexity. A recent application even accounts for site fidelity (Ditmer et al. 2023) that may emerge from preferences for some specific habitats.

## 5 Conclusion

The estimation of the strength of habitat selection for specific, easily measurable variables, such as vegetation cover types, is at the heart of the species distribution models on which we, ecologists, base our prediction of animal distribution under changing environments. Yet, we have shown here that bias in the estimation easily arises when one does not, or cannot, account for some “invisible” variables that affect the movement of animals, in particular familiarity with the place. We therefore urge researchers to go beyond the consideration of physical variables as predictors of movements (e.g., vegetation cover type, distance to features). Certainly, ecologists should strive to find ways to account for the effects of “invisible landscapes”, even if in imperfect ways. The importance of past experience of individuals, which is inaccessible to the human analyst using snapshot observation data of unknown individuals, calls for mechanistic distribution modeling integrating the cognitive processes underlying animal movement.

## Author Contributions

**Romain Dejeante:** conceptualization (supporting), formal analysis (lead), methodology (equal), writing – original draft (lead). **Rémi Lemaire‐Patin:** conceptualization (supporting), formal analysis (supporting), methodology (equal), supervision (supporting), writing – review and editing (supporting). **Simon Chamaillé‐Jammes:** conceptualization (lead), methodology (equal), supervision (lead), writing – review and editing (lead).

## Conflicts of Interest

The authors declare no conflicts of interest.

## Supporting information


Appendix S1.

Appendix S2.

## Data Availability

All data were simulated. Simulated data and codes used in this study are publicly available in a figshare repository (Dejeante, Lemaire‐Patin, and Chamaillé‐Jammes 2024a).
