# Supplementary material for: How Can Overlooking Social Interactions, Space Familiarity or Other “Invisible Landscapes” Shaping Animal Movement Bias Habitat Selection Estimations and Species Distribution Predictions?
Source: Ecol Evol. 2025 Jan 8;15(1):e70782. doi: 10.1002/ece3.70782 (PMC11707625; doi:10.1002/ece3.70782)
Supplement: Supplementary file 1 — Appendix S1. [file ECE3-15-e70782-s001.pdf]

Can overlooking 'invisible' landscapes bias habitat selection estimation and population distribution projections?

Romain Dejeante; Rémi Lemaire-Patin; Simon Chamaillé-Jammes

Appendix S1. Testing the influence of invisible landscapes on habitat selection analysis

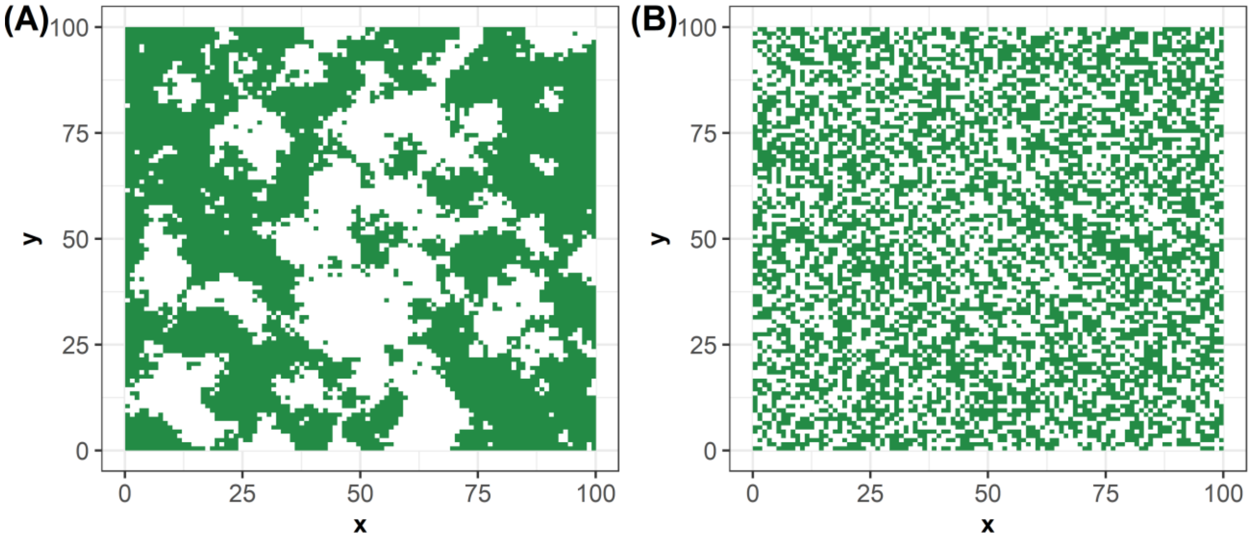

Figure S1. Example of landscapes simulated by discretizing spatially correlated Gaussian random fields. Green cells show the presence of the selected habitat-type (habitat A in main text). (A) More-patchy (ρ=10) and (B) less-patchy landscapes (ρ=1) were generated to test the influence of invisible landscape on habitat selection analysis.

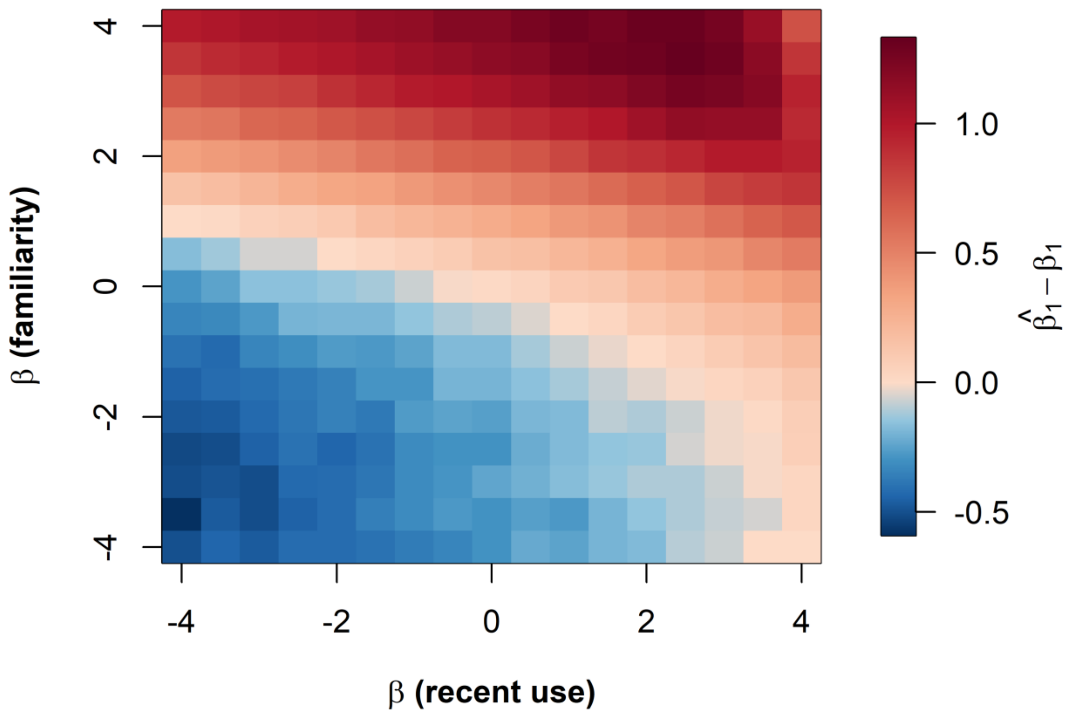

Figure S2. Influence of the strength of selection for familiar space ($\beta_{familiarity}$) and for recently-used areas ($\beta_{recent\ use}$) on habitat selection analyses. Cell values show the differences between the estimated ($\hat{\beta}_{habitat}$) and expected ($\beta_{habitat}$) coefficient of selection for habitat-type. Movement trajectories (n = 20 replications × 500 individuals ; step = 1000) were simulated on a fully parametrized LG movement model with coefficients ($\beta_{habitat} = 4$; $\beta_{familiarity}$; $\beta_{recent\ use}$).
