# Supplementary material for: How Can Overlooking Social Interactions, Space Familiarity or Other “Invisible Landscapes” Shaping Animal Movement Bias Habitat Selection Estimations and Species Distribution Predictions?
Source: Ecol Evol. 2025 Jan 8;15(1):e70782. doi: 10.1002/ece3.70782 (PMC11707625; doi:10.1002/ece3.70782)
Supplement: Supplementary file 2 — Appendix S2. [file ECE3-15-e70782-s002.pdf]

Can overlooking 'invisible' landscapes bias habitat selection estimation and population distribution projections?

Romain Dejeante; Rémi Lemaire-Patin; Simon Chamaillé-Jammes

Appendix S2. Testing the influence of invisible landscapes on population distribution predictions

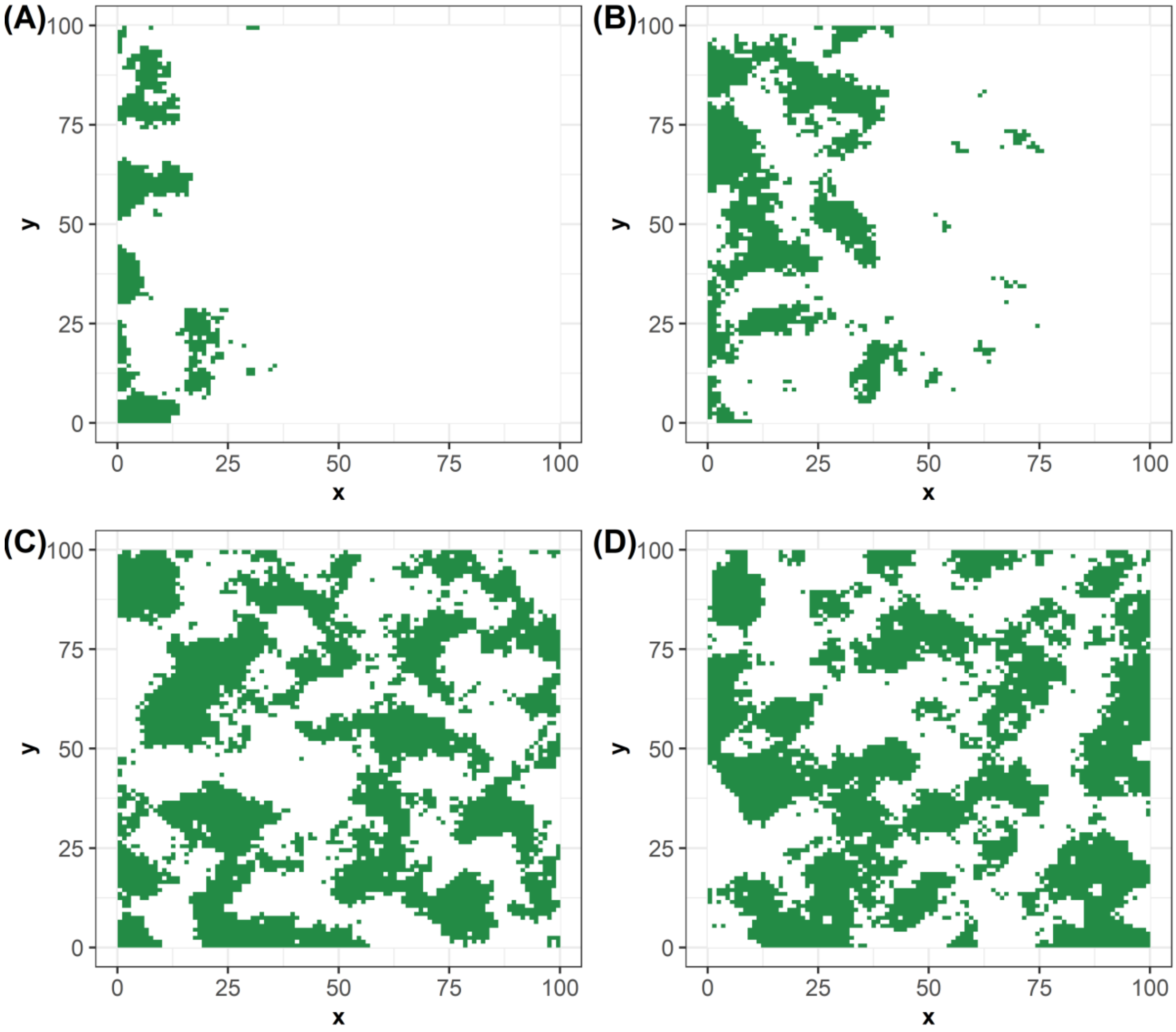

Figure S1. Example of landscapes simulated by discretizing spatially correlated Gaussian random fields, combined with planar gradient neutral landscapes with increasing proportions of the

selected habitat (habitat A, in green), to generate four successive landscapes over time, referred as (A) T0, (B) T1, (C) T2, (D) T3 in the result section.

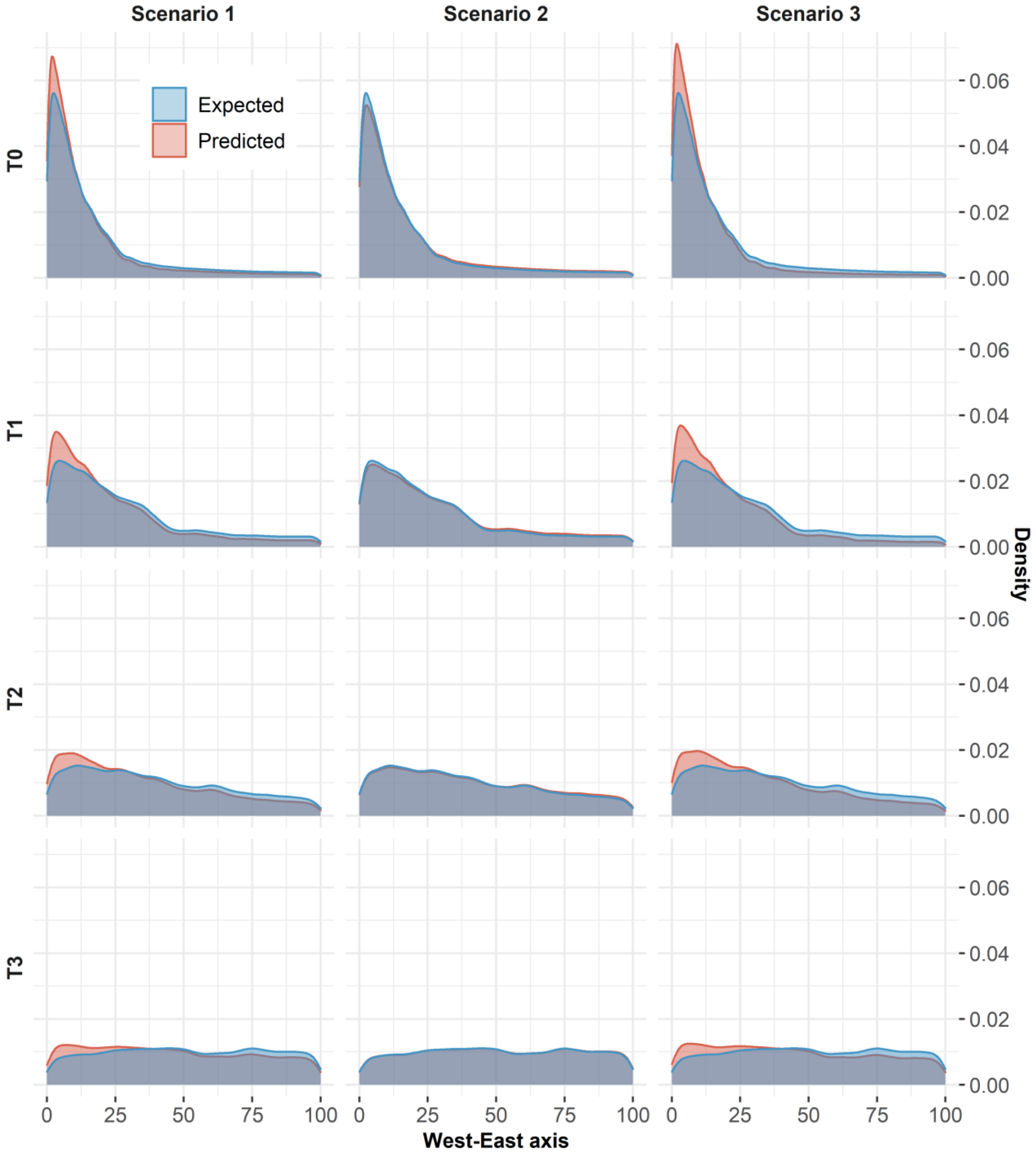

Figure S2. Population-distribution expected and predicted when simulated movements were independent on habitat familiarity ($\beta_{familiarity} = -4$) and recent use ($\beta_{recent\ use} = 0$).
